# Impact of HPV vaccination with Gardasil® in Switzerland

**DOI:** 10.1186/s12879-017-2867-x

**Published:** 2017-12-22

**Authors:** Martine Jacot-Guillarmod, Jérôme Pasquier, Gilbert Greub, Massimo Bongiovanni, Chahin Achtari, Roland Sahli

**Affiliations:** 1 Service of Gynecology and Obstetrics, Lausanne University Hospital, Pierre-Decker 2, 1011 Lausanne, Switzerland; 2 Institute of Social and Preventive Medicine, Lausanne University Hospital and University of Lausanne, Corniche 10, 1010 Lausanne, Switzerland; 3 Institute of Microbiology, Lausanne University Hospital, Bugnon 48, 1011 Lausanne, Switzerland; 4 Institute of Pathology, Lausanne University Hospital, Bugnon 25, 1011 Lausanne, Switzerland; 5 WHO HPV Regional Reference Laboratory for Europe, Bugnon 48, 1011 Lausanne, Switzerland

**Keywords:** Human papillomavirus, HPV genotyping, HPV vaccine, Gardasil®, *Chlamydia trachomatis*, Real-time PCR, PGMY-CHUV, Anyplex II™ HPV28, Segmented logistic regression, Cervical cancer screening

## Abstract

**Background:**

Gardasil®, a quadrivalent vaccine targeting low-risk (6, 11) and high-risk (16, 18) human papillomaviruses (HPV), has been offered to 11–14 year-old schoolgirls in Switzerland since 2008. To evaluate its success and its potential impact on cervical cancer screening, HPV genotypes were examined in 18-year-old girls five years later (sub-study 1) and in outpatients participating to cervical cancer screening before and after vaccine implementation (sub-study 2).

**Methods:**

For sub-study 1, 3726 females aged 18 in 2013 were invited to fill a questionnaire on personal demographics and HPV risk factors and to provide a self-collected cervicovaginal sample for HPV genotyping and *Chlamydia trachomatis* PCR. Personal data were evaluated by univariable and multivariable statistics. In sub-study 2, the proportion of the vaccine-type HPV among anogenital HPV was examined with archived genotyping data of 8039 outpatients participating to cervical cancer screening from 1999 till 2015. The yearly evolution of this proportion was evaluated by segmented logistic regression.

**Results:**

690 (18.5%) women participated to sub-study 1 and 327 (8.8%) provided a self-collected sample. Prevalence of *Chlamydia trachomatis* (4.6%) and demographics confirmed that the subjects were representative of sexually-active Swiss young women. Vaccine (five-year coverage: 77.5%) was preferentially accepted by contraceptive-pill users (*P* = 0.001) and samples were mainly provided by sexually-active subjects (*P* < 0.001). The proportion (4%) of the vaccine-type HPV in this population was lower than in sub-study 2 outpatients (*n* = 849, <26 years old) in the pre-vaccine era (25.7%). The proportion of the high-risk vaccine-type HPV decreased significantly (59%, *P* = 0.0048) in the outpatients during the post-vaccine era, yet this decrease was restricted to those aged less than 26 years (*n* = 673, *P* < 0.0001).

**Conclusions:**

The low proportion of vaccine-type HPV in 18-year-old females and its rapid decrease in young women participating to cervical cancer screening extend the success of HPV vaccination to Switzerland. Our data suggest that cervical cancer screening is now entering a stage of reduced proportion of HPV16 and/or 18 in samples reported positive by cytology. In view of the high likelihood of reduced clinical specificity of cytology, primary screening modalities involving HPV testing and cytology should now be re-evaluated in Switzerland.

**Electronic supplementary material:**

The online version of this article (10.1186/s12879-017-2867-x) contains supplementary material, which is available to authorized users.

## Background

Infection by high-risk (HR) anogenital human papillomaviruses (HPV) is a necessary cause of cervical cancer (CC) [1]. HPV16 and 18 account for 70% of cases worldwide, while the prototypic low-risk (LR) types, HPV6 and 11, cause the most frequent sexually-transmitted infection (condylomata acuminata). Acquisition of anogenital HPV is associated with sexual debut [2]. Most infections resolve within 2 years in immunocompetent individuals, and less than 1 % of initial HR HPV infections can progress to CC over an extended period of time (10–20 years) [3–5].

Vaccination against HR HPV targeting girls prior to sexual debut is expected to reduce CC burden, even in those who are not vaccinated thanks to herd immunity [6]. Three HPV vaccines are currently available: Cervarix® targeting HPV16 and 18, Gardasil® targeting HPV6, 11, 16, and 18, and Gardasil-9® targeting HPV6, 11, 16, 18, 31, 33, 45, 52, and 58. Cervarix® and Gardasil® could prevent 70% of CC cases and Gardasil-9® almost 90%. Proving HPV vaccines’ effectiveness against CC is difficult owing to the long delay between initial infection and cancer. Surrogate markers therefore have been proposed to determine vaccines’ effectiveness on a shorter term, such as population-based continuous monitoring of high grade precursor lesions (cervical intraepithelial lesions grade 3 or more, CIN3+) [7]. Accordingly, a CIN3+ obligatory reporting system is being evaluated for HPV vaccine monitoring in Switzerland [8].

Data obtained from large cohorts of women several years after implementation of Cervarix® [9, 10] or Gardasil® (reviewed by Garland et al. [11]) have shown that both vaccines are efficient at reducing the frequency of precursor lesions associated with the vaccine genotypes. However, even the nonavalent vaccine will not be able to prevent all CC cases, and a large fraction of older women are presently not vaccinated. Successful prevention of CC therefore will still rely on screening for years to come. Cervical cancer screening modalities will need to take into consideration the progressive reduction of abnormal lesions harbouring the most carcinogenic HPV genotypes. This reduction is expected to affect negatively the clinical specificity of cytology [12]. The knowledge of HPV burden in the transition from the pre-vaccine to the vaccine era therefore is important in countries such as Switzerland where cytology is used for primary screening and HPV testing as an adjunct in an opportunistic setting.

Gardasil® has been used in Switzerland and offered to schoolgirls aged 11–14 since 2008, yet its impact is not known in Switzerland. The aims of our work were to examine the proportion of vaccine-type HPV among anogenital HPV in sexually-active 18-year-old Swiss females five years after vaccine implementation, and the temporal impact of Gardasil® on this proportion in outpatients participating to CC screening from 1999 to 2015.

## Methods

### Study design

This is an observational study divided into two sub-studies aimed at evaluating the distribution of HPV genotypes within two sets of individuals in the context of Gardasil® implementation in Switzerland.

Sub-study 1 comprised 18-year-old females (*n* = 3726) invited through an independent marketing organization (BVA Logistique SA, Le Mont-sur-Lausanne, Switzerland) to participate in our study in 2013. BVA gets the residents’ records from all residents’ registration offices of the canton of Vaud. Sub-study-1 individuals corresponded to 82.2% of the 18-year-old women officially registered in the canton of Vaud as of December 31st 2013 (*n* = 4537, personal communication: Mabillard, H, October 2016, canton of Vaud Statistical Office) since residents can oppose their being included in the BVA database. Women of that age had all been targeted by the vaccination campaign initiated in 2008 when they were 13 years old, and could have accepted the vaccine within this five-year period because of catch-up vaccination that was offered to older girls aged up to 19. This group served to establish the proportion of vaccine-type HPV among anogenital-HPV in sexually-active young women, up to five years after having been offered HPV vaccination. It served also to establish the demographics and uptake of vaccination in young women in our canton to assess whether our findings can be generalized to Switzerland. They were sent a questionnaire by BVA to address (1) personal data, (2) preventive aspects pertaining to HPV infection and cervical cancer and (3) risk factors of HPV acquisition and of sexually transmitted infections (STI) (Additional file 1). The questionnaire served also for informed consent to provide a cervicovaginal sample for HPV genotyping and *Chlamydia trachomatis* (*CT*) testing, in which case women were sent a self-collection device to their home address that was encoded by BVA to ensure anonymity. *CT* prevalence was determined to evaluate the risk of STI independently of HPV vaccine acceptance because it had previously been assessed in sexually-active young women of the canton of Vaud to be used here as a comparator [13]. Vaud (*n* = 773′000 inhabitants) is one of 26 cantons of Switzerland (*n* = 8′327’000) and ranks third in terms of population after Zürich (*n* = 1′466’000) and Bern (n = 1′017’000). No financial incentive was used to encourage participation in the study. However free *CT* consultation was offered to those who were *CT*-positive provided they accepted to be contacted.

Sub-study 2 comprised all outpatients (*n* = 8039) who visited the clinics of the Gynaecology and Obstetrics Service of our hospital centre and affiliated family planning centres from March 1999 to December 2015. Sub-study 2 served to assess the evolution of the proportion of vaccine-type HPV encountered in screened women before (1999–2007) and after the introduction of HPV vaccination until 2015. HPV-genotype distribution was retrospectively assessed with archived genotyping data from a total of 12′706 samples submitted to our laboratory as an adjunct to abnormal cytology (*n* = 11′576) or as follow-up (FUP, n = 1′130) after Papanicolaou (PAP) testing. Abnormal cytology were atypical cells of undetermined significance (ASCUS, *n* = 6′983), low grade squamous intraepithelial lesions (LSIL, *n* = 4′423), and high grade squamous intraepithelial lesions (HSIL, *n* = 170). HPV testing of FUP samples was requested despite normal cytology for patients who presented previously with persistent ASCUS or to monitor treatment.

### HPV genotyping and *CT* real time PCR

Cervicovaginal sample (5 mL) was self-collected by sub-study-1 individuals with the Delphi Screener® device (Delphi Bioscience, BV Scherpenzeel, the Netherlands) and transferred in a coded tube prior to sending to our laboratory within 24 h. DNA from 200 μL cell suspension was isolated with the MagNaPure 96 Total Nucleic Acids kit according to the manufacturer (Roche, Basel, Switzerland) and eluted in 100 μL elution buffer. Five μL was used for *CT* detection by real time PCR [14] and for genotyping of 28 HPV genotypes (6, 11, 16, 18, 26, 31, 33, 35, 39, 40, 42–45, 51–54, 56, 58, 59, 61, 66, 68–70, 73, and 82) by the Anyplex™ II HPV28 kit and the HPV51 RUO kit according to the manufacturer (Seegene, Seoul, South Korea). The HPV51 RUO kit was used for HPV51, which is otherwise not well covered with the Anyplex™ II HPV28 kit [15]. HPV- or *CT-*negative tests were valid only if their internal positive control was positive (human DNA target and spiked internal control, respectively). Negative controls were tested in parallel throughout the procedures to monitor contaminations.

DNA extraction and HPV genotyping (PGMY-CHUV assay) for sub-study-2 individuals have been described [16, 17].

### Statistical analyses

Sub-study-1 questionnaires were reviewed to identify inconsistent responses. Together with empty fields, they were considered non informative (NI) and excluded from statistical analysis (Additional file 1). A final table was generated from the curated questionnaires and the microbiological data. Fisher’s tests have been used to highlight risk factors associated with HPV and *CT* infections. Multivariable logistic regression was used to investigate factors associated with vaccine acceptance and with self-sampling acceptance.

Sub-study-2 individuals were stratified in three age groups (< 26, 26–30, and >30) to chart the yearly proportion of the vaccine-type HPV among anogenital HPV. This proportion was expected to diminish in the youngest outpatients some year after vaccine introduction in 2008, and its evolution was analysed by segmented logistic regression according to the following model: logit(p) = b0 + b1 * year + b2 * max(year − A, 0), where p is the proportion of the vaccine HPV types among anogenital HPV and A is the breakpoint year at which the regression coefficient changed. The model before the breakpoint year A is logit(p) = b0 + b1 * year and the model after the breakpoint year A is logit(p) = (b0 − b2 * A) + (b1 + b2) * year. Hence the odds ratio associated with the yearly evolution of p before the breakpoint is exp(b1) while the odds ratio after the breakpoint is exp(b1 + b2) = exp(b1) * exp(b2). The value exp(b2) is the multiplicative coefficient at the breakpoint; it is a measure of the change in the odds ratio. A coefficient significantly different from 1 is indicative of a significant variation in the yearly evolution of p.

The age limit of 26 was based on the assumption that the majority of vaccinated women would be younger than 26, seven years after vaccine implementation, taking into account catch-up vaccination offered to women aged up to 19. Genotypes were stratified by year of sample collection and patient’s age. For each patient, only the first occurrence of a genotype was considered to avoid overrepresentation of persistent viruses and to enrich the dataset in incident infections. Analysis was restricted to the 26 anogenital HPV (6, 11, 16, 18, 26, 31, 33, 35, 39, 40, 42, 44, 45, 51–54, 56, 58, 59, 66, 68–70, 73, and 82) shared between PGMY-CHUV and Anyplex™ II HPV28 to facilitate comparison with the genotyping data of the 18-year-old sub-study-1 subjects. Version 1 of PGMY-CHUV was replaced mid 2013 by version 2 to improve detection of HPV68a [17]. HPV68a therefore was excluded from the calculation, which otherwise would have had artificially diminished the proportion of the four vaccine-type HPV from 2013 on.

Statistical analyses were performed with R version 3.2.3 [18] and GraphPad Prism (GraphPad Software, La Jolla, CA) using a significance level of 0.05. *P*-values were double-sided. Raw data and R script are available as Additional files 2 and 3, respectively.

## Results

Of 3726 sub-study-1 females, 690 (18.5%) responded to the questionnaire (Additional file 1). Most subjects reported being in good health condition (98.6%), using the contraceptive pill (64%) and being student or in apprenticeship (88.8%). The five-year vaccination coverage was 77.5%; 56.3% of the vaccinated subjects underwent the full three doses regimen. PAP test was normal for the majority of subjects (94.2%) who reported to have been examined (42.5%). All variables linked to undergoing sexual activity were associated with vaccine acceptance by univariable analysis (not shown). Being user of contraceptive pill (1.82, *P* = 0.001) and being in apprenticeship as opposed to the other two working status (student or other) (1.56, *P* = 0.04) were associated with a higher odds ratio by multivariable analysis (Table 1).

**Table 1:** Factors associated with vaccine acceptance in sub-study-1 participants

	Vaccination status Negative (*n* = 152)		Vaccination status Positive (*n* = 535)		Odds ratio			
Independent variable	Number	Proportion	Number	Proportion	value	95% CI		p-value
Contraceptive pill use Yes	80	0.53	361	0.67	1.82	1.26	2.63	< 0.001
Working status Apprentice	36	0.24	179	0.33	1.56	1.02	2.36	0.04

*CI* Confidence interval

Most (*n* = 562; 81.4%) of the 690 subjects had an intercourse at least once in the previous five years (mean age at sexual debut of 16.0 ± 1.4 years, Additional file 1) and less than 5 % reported sexual debut occurring before age 14. The number of sexual partners was one (*n* = 189; 33.6%), two to five (*n* = 240; 42.7%) and more than five (*n* = 62; 11%). Usage of condom was systematic for 115 subjects (20.5%). The majority of subjects had not been treated for STI (>95%), nine (1.3%) reported suffering from an immune-related disease and 203 reported being smokers (29.4%).

Five hundred and forty-nine (79.6%) subjects accepted self-sampling for HPV and *CT* analyses. Acceptance was found in association with “having sexual intercourse” (*P* < 0.001) and “being smoker” (*P* = 0.02) by multivariable analysis (Additional file 4, panel A). However, only 327 (59.6%) eventually provided a self-collected sample resulting in a low proportion (8.8%) of women among the entire population to be examined for HPV and *CT*. The working status “being student” was significantly associated with having actually provided a sample (*P* = 0.04) by multivariable analysis (Additional file 4, panel B). The vast majority (99.7%) considered the self-collection device user-friendly.

Of the 327 self-collected samples, 324 were informative for HPV and 327 for *CT*. The proportion of subjects infected by any type of HPV was 29.5% (2.8% for vaccine-type HPV only, Table 2). There were 38 single infections and 57 multiple infections with up to seven genotypes (average 3.1, not shown). *CT* infection was used as a proxy for risk of STI independent of HPV vaccination. The prevalence of *CT* infections was 4.6% (*n* = 15) and did not differ significantly with the vaccinal status of the subjects (Table 2). As expected the majority of *CT*-positive cases were found among HPV-positive women (13 *CT*-positive / 95 HPV-positive vs. 2 *CT*-positive / 229 HPV-negative, Fisher’s *P*-value <0.001).

**Table 2:** HPV and CT prevalence among sub-study-1 subjects

	Population (CT *n* = 327, HPV *n* = 324)			Vaccinees (CT *n* = 248, HPV *n* = 245)			Non vaccinees (CT *n* = 77, HPV n = 77)			p-value
	n	pr	sd	n	pr	sd	n	pr	sd	
CT	15	4.6	1.2	10	4.0	1.3	5	6.5	2.8	0.360
HPV global	95^a^	29.5	2.5	70	28.6	2.9	25	32.5	5.4	0.567
HPV vaccine	9^b^	2.8	0.9	6	2.4	1.0	3	3.9	2.2	0.451

Of 327 samples analysed, 327 were informative for *Chlamydia trachomatis* (CT) and 324 for Human papillomaviruses (HPV)

Two subjects were non informative regarding their vaccine status. They were CT and HPV negative. The 15 CT positive cases were found in 13 HPV positive cases and 2 HPV negative cases

n: number of positive subjects; pr: proportion in %; sd: standard deviation in %; *p*-value: two-sided Fishers’ *p* value

**Fig. 1:**
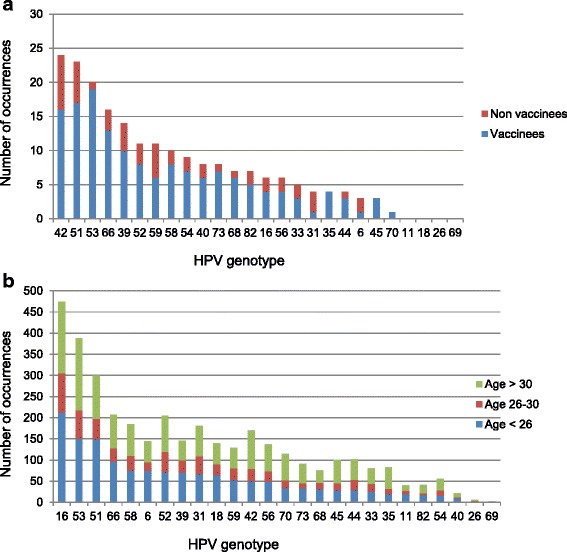
**a** Distribution of HPV genotypes in 95 sub-study-1 18-year-old girls in 2013, five years after having been offered Gardasil®. Genotypes were determined with the Anyplex™ II HPV28 kit and restricted to those shared with the PGMY-CHUV assay. The seven most prevalent genotypes by decreasing occurrences among vaccinees and non vaccinees were HPV42, 51, 53, 66, 39, 52 and 59. Members of the Gardasil® vaccine ranked 14th (HPV16), 20th (HPV6) and 23rd (HPV11 and 18). Vaccine did not affect the repartition of either genotypes among the non-vaccinated and vaccinated subjects (*p* > 0.05, not shown) although differences would be difficult to identify due to the low number of cases. The relatively high prevalence of HPV42 may in part be due to a higher sensitivity of Anyplex™ II HPV28 compared with PGMY-CHUV for this genotype. HPV genotyping with both assays is otherwise highly comparable [15]. **b** Distribution of HPV genotypes in 2272 HPV-positive sub-study-2 outpatients stratified in three age-groups referred to HPV testing from 1999 till 2007. Genotypes were determined with the PGMY-CHUV assay and restricted to those shared with the Anyplex™ II HPV28 kit. The seven most prevalent genotypes by decreasing occurrences among the youngest women (<26) were HPV16, 53, 51, 66, 58, 6, and 52. Members of the Gardasil® vaccine ranked first (HPV16), sixth (HPV6), 10th (HPV18) and 21st (HPV11)

^a^In total, 217 subject-genotype combinations were found in the 95 HPV positive subjects. Their distribution is shown in fig. 1

^b^Four subjects were infected by a single vaccine genotype. Five were infected by a single vaccine genotype and by at least another non vaccine genotype

Factors associated with HPV acquisition were being sexually active (*P* = 0.012) with a higher number of sex partners (*P* < 0.001) (Table 3). Condom use (*P* = 0.03), having been treated for an STI (*P* = 0.001), being examined with PAP (P = 0.001), and working status being neither a student nor an apprentice (P < 0.001) were also significantly associated with HPV positivity in contrast to tobacco smoking (*P* = 0.236). The working status was also associated with *CT* positivity (P = 0.01, not shown). The other univariable analyses for *CT* acquisition were however inconclusive because of the low number of positive cases.

**Table 3:** Factors affecting HPV positivity

	HPV neg	HPV pos	Proportion of HPV pos in %	Fisher test p-value
Sexually active				
No	24	2	7.7	0.012
Yes	198	91	31.5	
Number of sex partners				
1	78	14	15.2	< 0.001
2–5	86	32	27.1	
> 5	10	27	73.0	
Tobacco smoking				
No	163	61	27.2	0.236
Yes	66	34	34.0	
PAP smear				
No	133	37	21.8	0.001
Yes	92	57	38.3	
Condom use				
No	154	84	35.3	0.003
Yes	44	7	13.7	
Contraceptive pill use				
No	77	23	23.0	0.113
Yes	152	72	32.1	
STI treatment				
No	215	79	26.9	0.001
Yes	10	16	61.5	
Health status: good				
No	4	1	20.0	1.000
Yes	224	93	29.3	
Working status				
Student	148	40	21.3	< 0.001
Apprentice	64	33	34.0	
Other	17	22	56.4	

The sum of cases is not always equal to 324 because of non informative responses to the questionnaire, which have been excluded from analysis

*Neg* Negative, *Pos* Positive

The distribution of the 217 genotype-sample combinations in 18-year-old sub-study-1 subjects in 2013 is graphically displayed according to their vaccination status in Fig. 1 a. The proportion of the four vaccine-type HPV (*n* = 9) was 4% (9/217), with HPV16 (*n* = 6) ranking 14th, HPV6 (*n* = 3) 20th, HPV11 and HPV18 (*n* = 0) 23rd.

**Fig. 2:**
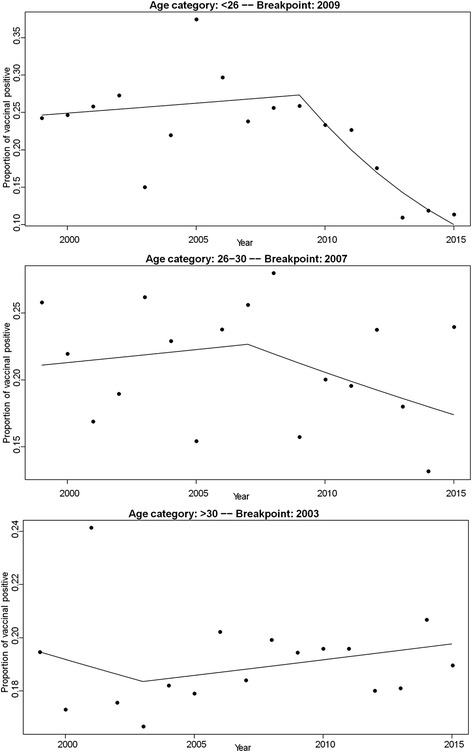
Segmented logistic regression of the yearly proportion of vaccine-type HPV in 3869 HPV-positive sub-study-2 outpatients from 1999 till 2015. Genotypes were determined with the PGMY-CHUV assay and corrected for HPV68a bias (see Methods), and restricted to those 26 shared with the Anyplex™ II HPV28 kit. A significant (*p* < 0.001) reduction of the yearly proportion of the four vaccine-type HPV among shared HPV genotypes is evident only in the outpatients aged <26 after the breakpoint year (2009). For the other age groups the breakpoint year was not associated with a significant variation of the odds ratio (*p* > 0.2) (Additional file 5)

The HPV genotype-sample combinations (*n* = 3619) observed before vaccine implementation (1999–2007) in the sub-study-2 outpatients were distributed by year and patients’ age (<26, 26–30 and >30), and ranked by decreasing occurrences within the youngest age group (<26, *n* = 1438 genotype-sample combinations) (Fig. 1 b). The proportion of the four vaccine-type HPV (*n* = 369) was 25.7%, with HPV16 (*n* = 212) ranking first, HPV6 (*n* = 74) fifth together with HPV58, HPV18 (*n* = 64) 10th, and HPV11 (*n* = 19) 21st. Restricting the outpatients’ population to the youngest age (<26) with normal cytology (*n* = 87; 50 HPV-negative) gave a similar proportion (25%) (n = 14 out of 56 genotype-sample combinations); HPV16 (n = 7) ranked first (data not shown). To test whether this proportion decreased after vaccine implementation, as suggested by the low proportion observed in the sub-study-1 subjects, the yearly proportion of the four vaccine-type HPV was calculated from 1999 to 2015 for each of the three age groups of outpatients, and its evolution examined by segmented logistic regression. Model adjustment was made to evaluate the year at which the regression coefficient of the model changed (breakpoint year, Additional file 5). No significant variation was observed for the patients aged >25. In contrast, a significant reduction in the proportion of the four vaccine-type HPV was observed for the patients aged <26 starting in 2010, two years after the vaccination campaign was initiated (breakpoint year 2009, OR = 0.82, *P* < 0.0001) (Fig. 2 and Additional file 5). The same analysis performed after further stratifying the youngest age group (<21, 21–25) confirmed this result (OR = 0.73, *P* = 0.05 and OR = 0.77, *P* < 0.001, respectively; Additional file 5). To establish the percentage of reduction of the vaccine-type HPV, the average yearly proportions of the vaccine-type HPV in the pre-vaccine era (1999–2007) was compared with that in the post-vaccine era at the plateau (2013–2015, fig. 2) for the youngest outpatients (<26). It was 25.5% (95% CI: 20.8%–30.1%) in the pre-vaccine era and significantly reduced by 56% (*P* = 0.0028 unpaired t test) in the post-vaccine era to 11.3% (95% CI: 10.2%–12.5%). Restricting the vaccine genotypes to HPV16 and HPV18, excluding HPV6 and HPV11 from the calculations, gave similar results; average yearly proportions of 21.0% (95% CI: 16.6%–25.3%) compared with 8.7% (95% CI: 6.7%–10.8%), 59% reduction (*P* = 0.0048).

## Discussion

This study is the first to investigate the impact of Gardasil® after its introduction in 2008 in Switzerland. We first assessed vaccination coverage and demographics as well as HPV genotypes in an 18-year-old women population targeted by the vaccine five years earlier in the canton of Vaud. The participation rate was low with 18.5% returned questionnaires and 8.8% self-collected samples. The low participation rate is a major weakness of this sub-study that necessitated to verify the representativeness of the participants. Reassuringly, our sub-study population was comparable to the Swiss women population of similar age based on risk factors for HPV such as hormonal contraception and use of condoms (Swiss Federal Statistical Office) [19] and on demographics of adolescents in Switzerland (SMASH-02 report) [20]. The education levels of the participants were also comparable to the general population of the same age in the canton of Vaud. Thus the proportions of students (57.5% ± 3.7%) and subjects without formation (11.1% ± 2.3%) overlapped those of the 18-year-old population registered December 31st 2013 (53.1% ± 1.5%, 8.8% ± 0.8%, respectively) while apprentices (31.3% ± 3.5% vs. 38.0% ± 1.4%, respectively) were slightly underrepresented (personal communication: Mabillard, H, October 2016, canton of Vaud Statistical Office). Vaccinees and non vaccinees showed similar rates of *CT* infections, consistent with HPV vaccination not influencing sexual behaviour as reported by others in England [21] and in Canada [22]. In addition the *CT* prevalence rate (4.6 ± 1.2%) was similar (5.5 ± 0.9%) to that of a previous survey of sexually-active women aged <25 in the same area of Switzerland [13], consistent with sexually-active subjects having preferentially provided a self-collected sample.

In the canton of Vaud, 69.5% of the schoolgirls aged 14 have been vaccinated (≥1 dose) against HPV during the 2010–2011 school year [23]. The vaccination rate reported by the subjects in our study is somewhat higher at 77.5%. This is most likely due to catch-up vaccination offered to women aged up to 19. In contrast, only 56% of the vaccinees reported having had the three-dose regimen compared with 96% in the 2010–2011 survey. This difference most likely reflects some degree of unreliability of the self-reported vaccination status (regimen in particular). This mode of reporting was preferred to consultation of vaccination books by nurses to minimize costs and ensure anonymous treatment of the files according to our ethical approval.

The percentages of women who systematically used condoms (20.5%) and contraceptive pills (64%) are similar to those of young women in a stable relationship (36.1% and 64%, respectively) (SMASH-02 report) [20]. This suggests that the majority was engaged in a stable relationship at time of questionnaire, consistent with the mean age at sexual debut (16.0 ± 1.4 years). This age is similar to that reported in Germany and in other European countries [24–26]. In addition, less than 5% of girls from our study reported having initiated sexual intercourse at 13 years or younger, in line with the recommended age (11–14) for vaccination in Switzerland.

A low percentage of returned samples (12%) was reported in a German study assessing HPV prevalence in women aged 20–25 years using the Delphi-Screener® [24]. The even lower percentage (8.8%) in our study may reflect consecutive shipment of questionnaire and self-collection devices while it was simultaneous in the German study. Self-sampling was accepted more frequently by women having sexual intercourse, and those actually providing a sample were more frequently students. This latter observation may be related to a possibly better knowledge of HPV infection by women with a higher level of education.

Global HPV prevalence increased with number of sex partners and other variables linked to undergoing sexual activity, as well as with lower degree of education. These data confirm previously published data on identified risk factors for HPV infection [4]. Four participants in the sub-study-1 vaccinated group were positive for HPV16. Three reported to have received the three recommended doses, whereas one mentioned two doses. All four reported being sexually active after the vaccination was completed. The low response rate and the overall low numbers of vaccine-type HPV-positive cases as well as some degree of unreliability of self-reporting did not allow meaningful statistical evaluation of the age at vaccination and age at first sex to test the hypothesis that HPV16-positive vaccinees may have been infected before vaccination. Taking into account the information provided by the volunteers, the presence of HPV16 in vaccinees may also reflect transient infections or vaccine failures that cannot be distinguished since HPV genotyping was performed at a single time point, thereby excluding assessment of persistent infections.

In view of the vaccination coverage well above 50%, the low proportion (4%) of the vaccine-type HPV was expected in our 18 year-old subject population compared with that (25%) in the youngest sub-study-2 outpatients during the pre-vaccination era according to a recent meta-analysis of the impact of HPV vaccination programmes and herd effect [6]. Herd immunity induced by HPV vaccination is reported by more and more researchers across the globe [11]. For instance, several studies have shown a major decline in the prevalence of vaccine-type HPV and in clinical expressions of HPV-induced illnesses like genital warts in Australian boys before their access to HPV vaccination [27].

The lower proportion of the vaccine-type HPV in sub-study-1 subjects may have resulted from using different means of sample collection (Delphi screener self-collection vs. PAP smear) and HPV genotyping assays (Anyplex II™ HPV28 vs. PGMY-CHUV), hence for this reason was not tested statistically. The strength of our study however relied on our ability to assess without bias the evolution of the yearly proportion of the vaccine-type HPV with the sub-study-2 outpatients’ data because the cervical cancer screening algorithm, sample collection and HPV genotyping method remained identical over the years (1999–2015). The youngest sub-study-2 outpatients (<26) were most likely enriched in women to whom the vaccine was offered since 2008, while the older women (26–30 and >30) were gradually less likely to have received the vaccine or have benefitted from herd immunity. Consistent with this and with the known clearance time of HPV (90% within two years), a time wise significant reduction of the yearly proportion of the four vaccine-type HPV was observed for our outpatients aged <26. The 56% reduction of the four vaccine-type HPV and the 59% reduction of HPV16 and HPV18 achieved as of five years after vaccine implementation is reminiscent of the numbers published in the US [28] [29] and in the UK [30–32]. Our data add to these studies the yearly kinetic of reduction of the vaccine-type HPV in a real-world condition of opportunistic cervical cancer screening. We could not establish the prevalence of HPV genotypes in the global population since the sub-study-2 population represented a high-risk group of screened women referred to HPV testing based on cervical abnormalities. Also, we could not evaluate with confidence the influence of vaccination on the proportion of high grade lesions, population wise, as has been published for Scotland, which shows a reduction in the proportion of CIN lesions in vaccinees under an organized screening algorithm [33]. Our data will however be useful to evaluate cervical cancer screening modalities in the vaccine era in Switzerland and in other countries with similar vaccination coverage and opportunistic screening algorithms.

## Conclusions

The low proportion of vaccine-type HPV in 18-year-old females and its rapid decrease in young women participating to cervical cancer screening extend the success of HPV vaccination to Switzerland. Our data suggest that cervical cancer screening is now entering a stage of declining prevalence of precursor lesions associated with the two most carcinogenic HPV as proposed earlier by a modelisation study [34]. There is good evidence that the decline of HPV16 and HPV18 will negatively affect the clinical specificity of cytology but not that of high-risk HPV testing [12] with a concomitant diminution of the positive-predictive value of cytology [35]. Primary screening modalities involving HPV testing and cytology should now be re-evaluated in Switzerland.

## Additional files

Additional file 1: Sub-study-1 flowchart and questionnaire results. (PDF 418 kb)

Additional file 2: Incidence of HPV genotypes by age and age categories observed in sub-study-2 outpatients participating to cervical cancer screening between 1999 and 2015. This file should be renamed “HPV_1999–2015_evolution_raw_data.xlsx” to be used by the R script provided as Additional file 3. (XLSX 221 kb)

Additional file 3: R script used to graph the yearly evolution of the vaccine-type HPV proportion recorded in the Additional file 2. It can be renamed “HPV_1999–2015_evolution.R”. (R 3 kb)

Additional file 4: Factors associated with self-sampling acceptance and providing a sample by sub-study-1 participants. (DOCX 16 kb)

Additional file 5: Segmented logistic regression analysis of the evolution of the yearly proportion of vaccine-type HPV (vHPV) among all HPV in sub-study-2 outpatients. (DOCX 27 kb)

## Abbreviations

ASCUS: Atypical cells of undetermined significance
BVA: Bureau vaudois d'adresses
CC: Cervical cancer
CI: 95% confidence interval
CIN: Cervical intraepithelial neoplasia
*CT*: *Chlamydia trachomatis*
FUP: Follow-up
HPV: Human papillomavirus
HR: High-risk
HSIL: High grade squamous intraepithelial lesions
LR: Low-risk
LSIL: Low grade squamous intraepithelial lesions
NI: Non informative
OR: Odds ratio
PAP: Papanicolaou testing
PCR: Polymerase chain reaction
STI: Sexually transmitted infection
