# Supplementary material for: Impact of HPV vaccination with Gardasil® in Switzerland
Source: BMC Infect Dis. 2017 Dec 22;17:790. doi: 10.1186/s12879-017-2867-x (PMC5741926; doi:10.1186/s12879-017-2867-x)
Supplement: Supplementary file 1 — Sub-study-1 flowchart and questionnaire results. (PDF 418 kb) [file 12879_2017_2867_MOESM1_ESM.pdf]

Additional file 1. Sub-study-1 flowchart and questionnaire results.

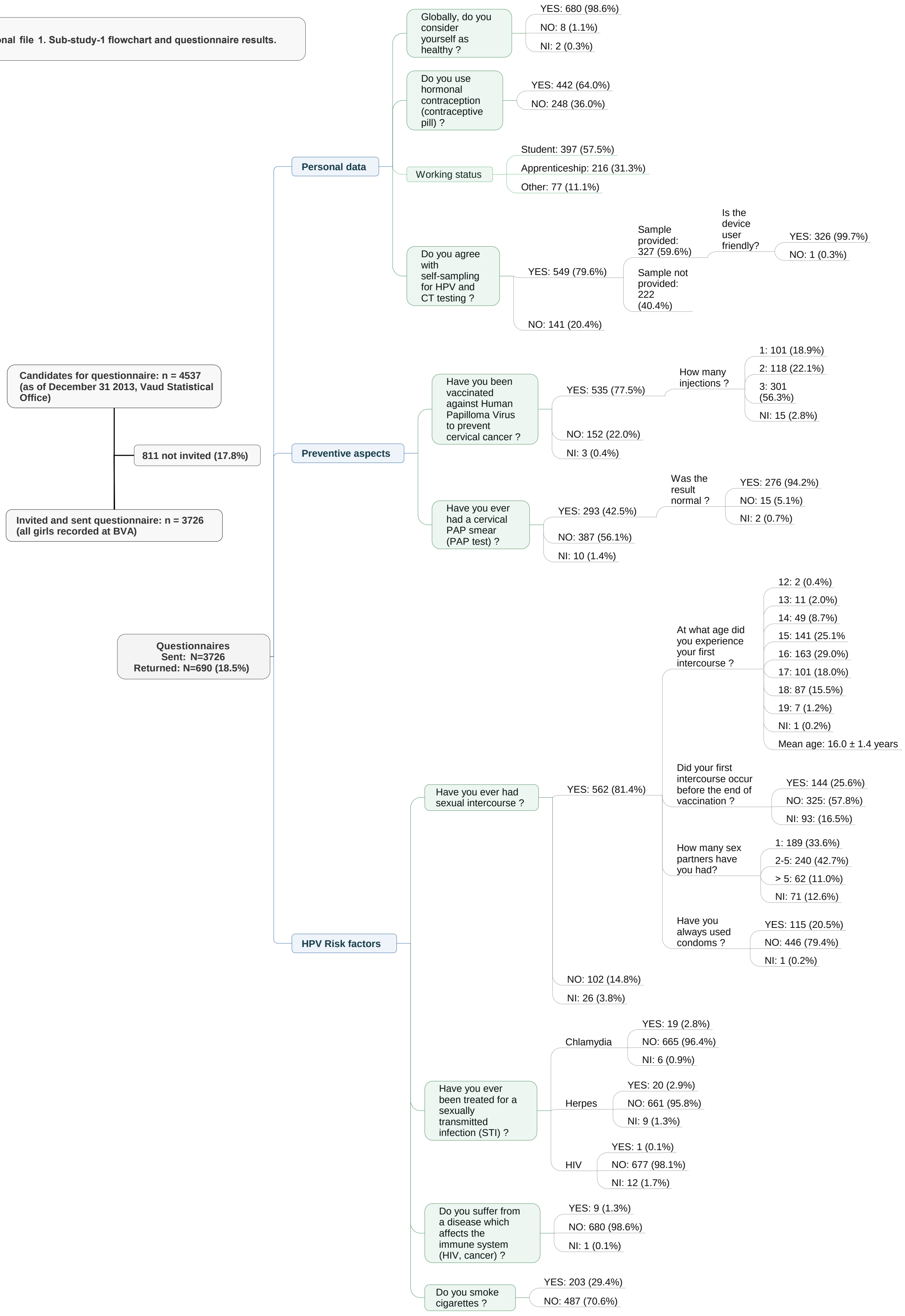

Additional file 1. Sub-study-1 flow chart and questionnaire results.

BVA: Bureau vaudois des adresses. This is an independent marketing organization that registers all individuals who agree to be contacted for any issue.
NI: non informative responses that were excluded from analysis (empty fields in questionnaires for specific items, inconsistent response such as not reporting PAP yet reporting a normal PAP result)
HPV: Human papillomavirus; CT: *Chlamydia trachomatis;* PAP: cervical smear stained with Papanicolaou reagent for cervical cancer screening; HIV: Human immunodeficiency virus
