# Supplementary material for: Impact of HPV vaccination with Gardasil® in Switzerland
Source: BMC Infect Dis. 2017 Dec 22;17:790. doi: 10.1186/s12879-017-2867-x (PMC5741926; doi:10.1186/s12879-017-2867-x)
Supplement: Supplementary file 4 — Factors associated with self-sampling acceptance and providing a sample by sub-study-1 participants. (DOCX 16 kb) [file 12879_2017_2867_MOESM4_ESM.docx]

**Additional file 4. Factors associated with self-sampling acceptance and providing a sample by sub-study-1 participants.**

A: Self sampling acceptance

|  | **Refuse (n = 132)** | | **Accept (n = 524)** | | **Odds ratio** | | | |
| --- | --- | --- | --- | --- | --- | --- | --- | --- |
| **Independent variable** | **Number** | **Proportion** | **Number** | **Proportion** | **OR** | **95% confidence interval** | | **p-value** |
| Sexual intercourse  Yes | 78 | 0.59 | 477 | 0.91 | 8.26 | 4.78 | 14.28 | < 0.001 |
| Smoker  Yes | 24 | 0.18 | 175 | 0.33 | 1.82 | 1.10 | 3.02 | 0.02 |
| PAP  Yes | 50 | 0.38 | 241 | 0.46 | 0.62 | 0.38 | 1.01 | 0.05 |
| Working status  Other | 7 | 0.05 | 66 | 0.13 | 2.10 | 0.90 | 4.87 | 0.08 |

n = 656. Thirty four observations were excluded from analysis because of missing values in one of the addressed variables for 34 subjects among the 690 who responded to the questionnaire.

B: Having actually provided a self-collected sample

|  | **Sample not provided (n = 207)** | | **Sample provided (n = 308)** | | **Odds ratio** | | | |
| --- | --- | --- | --- | --- | --- | --- | --- | --- |
| **Independent variable** | **Number** | **Proportion** | **Number** | **Proportion** | **OR** | **95% confidence interval** | | **p-value** |
| STI treatment  HSV | 4 | 0.02 | 13 | 0.04 | 1.93 | 0.60 | 6.17 | 0.27 |
| Working status  Student | 106 | 0.51 | 180 | 0.58 | 1.47 | 1.02 | 2.11 | 0.04 |
| Unprotected sex | 143 | 0.69 | 232 | 0.75 | 1.27 | 0.84 | 1.92 | 0.25 |
| PAP  No | 1 | 0.00 | 9 | 0.03 | 7.13 | 0.87 | 58.27 | 0.07 |
| PAP  Yes | 86 | 0.42 | 140 | 0.45 | 1.18 | 0.81 | 1.72 | 0.39 |

n = 515. Thirty four observations were excluded from analysis because of missing values in one of the addressed variables for 34 subjects among the 549 who initially accepted to provide a self-collected sample.
