# Supplementary material for: Impact of HPV vaccination with Gardasil® in Switzerland
Source: BMC Infect Dis. 2017 Dec 22;17:790. doi: 10.1186/s12879-017-2867-x (PMC5741926; doi:10.1186/s12879-017-2867-x)
Supplement: Supplementary file 5 — Segmented logistic regression analysis of the evolution of the yearly proportion of vaccine-type HPV (vHPV) among all HPV in sub-study-2 outpatients. (DOCX 27 kb) [file 12879_2017_2867_MOESM5_ESM.docx]

**Additional file 5. Segmented logistic regression analysis of the evolution of the yearly proportion of vaccine-type**

**HPV (vHPV) among all HPV in sub-study-2 outpatients.**

| **Age Category** | **Breakpoint** | **Before breakpoint** | | | | **Multiplicative coefficient at the breakpoint** | | | | **After breakpoint** | | | |
| --- | --- | --- | --- | --- | --- | --- | --- | --- | --- | --- | --- | --- | --- |
|  |  | **OR** | **CI 95%** | | **p-value** | **Coeff** | **CI 95%** | | **p-value** | **OR** | **CI 95%** | | **p-value** |
| <21 | 2010 | 1.05 | 1.01 | 1.10 | 0.03 | 0.70 | 0.48 | 0.95 | 0.03 | 0.73 | 0.52 | 0.98 | 0.05 |
| 21-25 | 2011 | 0.98 | 0.95 | 1.01 | 0.11 | 0.78 | 0.66 | 0.92 | <0.005 | 0.77 | 0.65 | 0.89 | <0.001 |
| <26 | 2009 | 1.01 | 0.98 | 1.04 | 0.35 | 0.81 | 0.73 | 0.89 | <0.0001 | 0.82 | 0.75 | 0.88 | <0.0001 |
| 26-30 | 2007 | 1.01 | 0.95 | 1.07 | 0.70 | 0.95 | 0.85 | 1.06 | 0.35 | 0.96 | 0.90 | 1.02 | 0.22 |
| >30 | 2003 | 0.98 | 0.89 | 1.09 | 0.73 | 1.03 | 0.91 | 1.16 | 0.68 | 1.01 | 0.98 | 1.04 | 0.63 |

Breakpoint: year at which the regression coefficient changed in the model functions used to represent the yearly evolution of the proportion of vHPV; OR: odds ratio; CI: confidence interval; Coeff = exp(b2): multiplicative coefficient of the OR at the breakpoint (see Methods in the main manuscript).
